# Risk prediction models for enteral feeding intolerance in critically ill patients: a systematic review

**DOI:** 10.3389/fpubh.2026.1900308

**Published:** 2026-09-09

**Authors:** Yuting Xie, Chunxia Li, Xijun Wu, Chunyan Wang

**Affiliations:** Department of Critical Care Medicine, West China Hospital, Sichuan University/West China School of Nursing, Sichuan University, Chengdu, Sichuan, China

**Keywords:** critically ill patients, enteral nutrition, feeding intolerance, prediction model, systematic review

## Abstract

**Objective:**

To systematically evaluate existing risk prediction models for enteral feeding intolerance (EFI) in critically ill patients, with the aim of providing evidence-based references for clinical practice.

**Methods:**

PubMed, Web of Science, the Cochrane Library, China National Knowledge Infrastructure (CNKI), Wanfang Database, China Biology Medicine Disc (CBM), and VIP Database were systematically searched from database inception to November 20, 2025. Studies that developed or validated risk prediction models for enteral feeding intolerance in adult critically ill patients were included. Data extraction was conducted using the CHARMS checklist, and the risk of bias and applicability of the included studies were assessed using the PROBAST tool.

**Results:**

A total of 25 studies were included, involving 25 risk prediction models for enteral feeding intolerance. Sample sizes ranged from 118 to 1,584 patients, and the incidence of EFI ranged from 26.25 to 61.3%. APACHE II score, blood glucose, mechanical ventilation, age, albumin, and intra-abdominal pressure were relatively frequently reported predictors, but no single predictor was consistently identified across all models.

**Conclusion:**

Existing risk prediction models for enteral feeding intolerance in critically ill patients demonstrate certain predictive value; however, the methodological quality varies considerably, and external validation is insufficient. Future studies should conduct large-scale, multicenter, prospective research and adopt standardized methodological procedures to develop and validate prediction models, thereby improving their clinical applicability and generalizability.

**Systematic review registration:**

https://www.crd.york.ac.uk/PROSPERO/view/CRD420261281618, identifier (CRD420261281618).

## 1 Introduction

Critically ill patients, due to severe infections, trauma, shock, and multiple organ dysfunction, are often in a hypermetabolic and hypercatabolic state, resulting in a significantly increased incidence of malnutrition (1). Studies have shown that malnutrition is closely associated with adverse outcomes in critically ill patients, including increased infection rates, prolonged duration of mechanical ventilation and ICU stay, and even increased mortality (2). Therefore, early and appropriate nutritional support has become an essential component of comprehensive treatment in critically ill patients.

Enteral nutrition (EN), which follows physiological pathways, helps maintain intestinal mucosal barrier function, modulate immune responses, and reduce infectious complications (3). Consequently, it is recommended by multiple national and international guidelines as the preferred nutritional support strategy for critically ill patients (4). International and Chinese guidelines recommend initiating enteral nutrition as early as possible in hemodynamically stable patients to improve clinical outcomes (3–5). However, in clinical practice, enteral feeding intolerance (EFI) is common among critically ill patients, with reported incidence rates ranging from 20 to 60%, varying widely across studies (6, 7). Feeding intolerance typically manifests as increased gastric residual volume, abdominal distension, vomiting, diarrhea, reflux, and aspiration, which may lead to interruption of enteral nutrition, insufficient actual energy intake, and increased risks of aspiration pneumonia, infection, and mortality (8, 9).

To reduce the risk of enteral feeding intolerance, researchers have attempted to develop risk prediction models to estimate the probability of feeding intolerance in critically ill patients (10). These models are usually based on demographic characteristics, disease severity scores, hemodynamic status, medication use, and gastrointestinal function indicators, and are constructed using statistical methods such as logistic regression. Model performance is commonly evaluated using discrimination, calibration, and validation measures (11). A multicenter cluster-randomized controlled trial has been designed to evaluate whether a prediction-model-guided feeding protocol can improve enteral nutrition management and patient outcomes; however, clinical effectiveness data are not yet available (10).

However, current studies on risk prediction models for enteral feeding intolerance in critically ill patients show substantial heterogeneity in study populations, outcome definitions, predictor selection, and model validation methods. Therefore, the predictive performance and clinical applicability of these models require systematic evaluation. A comprehensive systematic review of existing studies is necessary.

Based on this background, the present study aims to systematically summarize the current status of research on risk prediction models for enteral feeding intolerance in critically ill patients, analyze model development methods, predictive factors, and predictive performance, evaluate the risk of bias and clinical applicability, and provide evidence-based guidance for clinical practice and future research.

## 2 Materials and methods

### 2.1 Literature inclusion and exclusion criteria

*Inclusion criteria*: (1) Adult critically ill patients receiving enteral nutrition; (2) Studies focused on the development or validation of risk prediction models for enteral feeding intolerance in critically ill patients; (3) Study designs including cohort studies, cross-sectional studies, and case–control studies; (4) Articles published in Chinese or English. *Exclusion criteria*: (1) Studies analyzing only risk factors without establishing a prediction model; (2) Incomplete data or inaccessible full texts; (3) Duplicate publications; (4) Conference abstracts; (5) Studies constructing models based on systematic reviews or meta-analyses. This systematic review has been registered with PROSPERO (registration ID: CRD420261281618).

### 2.2 Literature search strategy

PubMed, Web of Science, the Cochrane Library, CNKI, Wanfang Database, China Biology Medicine Disc (CBM), and VIP Database were systematically searched for studies on the development and validation of risk prediction models for enteral feeding intolerance in critically ill patients. The search period covered database inception to November 20, 2025, with publications limited to Chinese or English. Both subject headings and free-text terms were used, supplemented by manual searching of reference lists. Search terms included: Critical Illness, Intensive Care Units, ICU, Critical Care, Enteral Nutrition, tube feeding, gastric feeding, postpyloric feeding, Gastrointestinal Intolerance, Gastrointestinal Diseases, Feeding Intolerance, Risk Prediction Models, Risk Assessment, Predict*, Prediction Model.

This systematic review adopted the PICOTS framework recommended by the CHARMS checklist to define evaluation objectives, search strategies, and inclusion/exclusion criteria:

- P (Population): Adult critically ill patients admitted to intensive care units and receiving enteral nutrition.
- I (Index prognostic model): Prediction models developed to estimate the risk or probability of enteral feeding intolerance.
- C (Comparative model): No competing model.
- O (Outcome): Gastrointestinal symptoms or events leading to interruption or inadequate delivery of enteral nutrition.
- T (Timing): Before or at initiation of enteral nutrition, with outcomes assessed during ICU stay.
- S (Setting): Intensive care units in hospital settings.

### 2.3 Literature screening and data extraction

Note Express software was used to remove duplicate records. Two researchers trained in evidence-based methodology independently screened titles and abstracts, followed by full-text review based on inclusion and exclusion criteria. Discrepancies were resolved through discussion or consultation with a third researcher.

Data extraction was guided by the CHARMS checklist, developed by Altman and Moons in 2014 (11). Extracted information included first author, study design, publication year, data source period, modeling method, validation method, predictive factors, and model performance (12). Data extraction was conducted independently by two researchers, with disagreements resolved by a third researcher. For continuous and dynamically changing predictors, we further extracted whether the original studies reported measurement timing, modeling form, cut-off values, threshold-selection methods, or assessment of nonlinear/time-dependent effects. We also extracted detailed components of EFI definitions, including whether GRV was used, GRV thresholds, measurement timing, whether GRV was assessed as a single measurement or cumulative 24-h volume, and the presence of other clinical manifestations such as vomiting, reflux, diarrhea, abdominal distension, aspiration, gastrointestinal bleeding, bowel sound changes, and failure to achieve target energy intake. In addition, we extracted whether the included studies evaluated model-guided clinical interventions or implementation effects.

### 2.4 Risk of bias and applicability assessment

The risk of bias and applicability of the included studies were assessed using the Prediction model Risk Of Bias ASsessment Tool (PROBAST) developed by Wolff et al. in 2019 (13). The tool evaluates the risk of bias across four domains, including participants, predictors, outcomes, and statistical analysis, and assesses applicability across three domains, namely participants, predictors, and outcomes. Each signaling question in the PROBAST tool is answered as “yes,” “probably yes,” “probably no,” “no,” or “no information,” and domain-level judgments of risk of bias are classified as low, high, or unclear based on the responses. If one or more domains are judged as having a high risk of bias, the overall risk of bias of the study is considered high. Two researchers independently conducted the risk of bias and applicability assessment for all included studies using the PROBAST tool (14, 15). In cases of disagreement, a third researcher was consulted, and consensus was reached through discussion. The assessment results were summarized and presented in tabular form, including both domain-specific judgments and overall judgments of risk of bias and applicability for each included study, and the detailed results are shown in Supplementary Table S3.

## 3 Results

### 3.1 Literature search process

**Figure 1:**
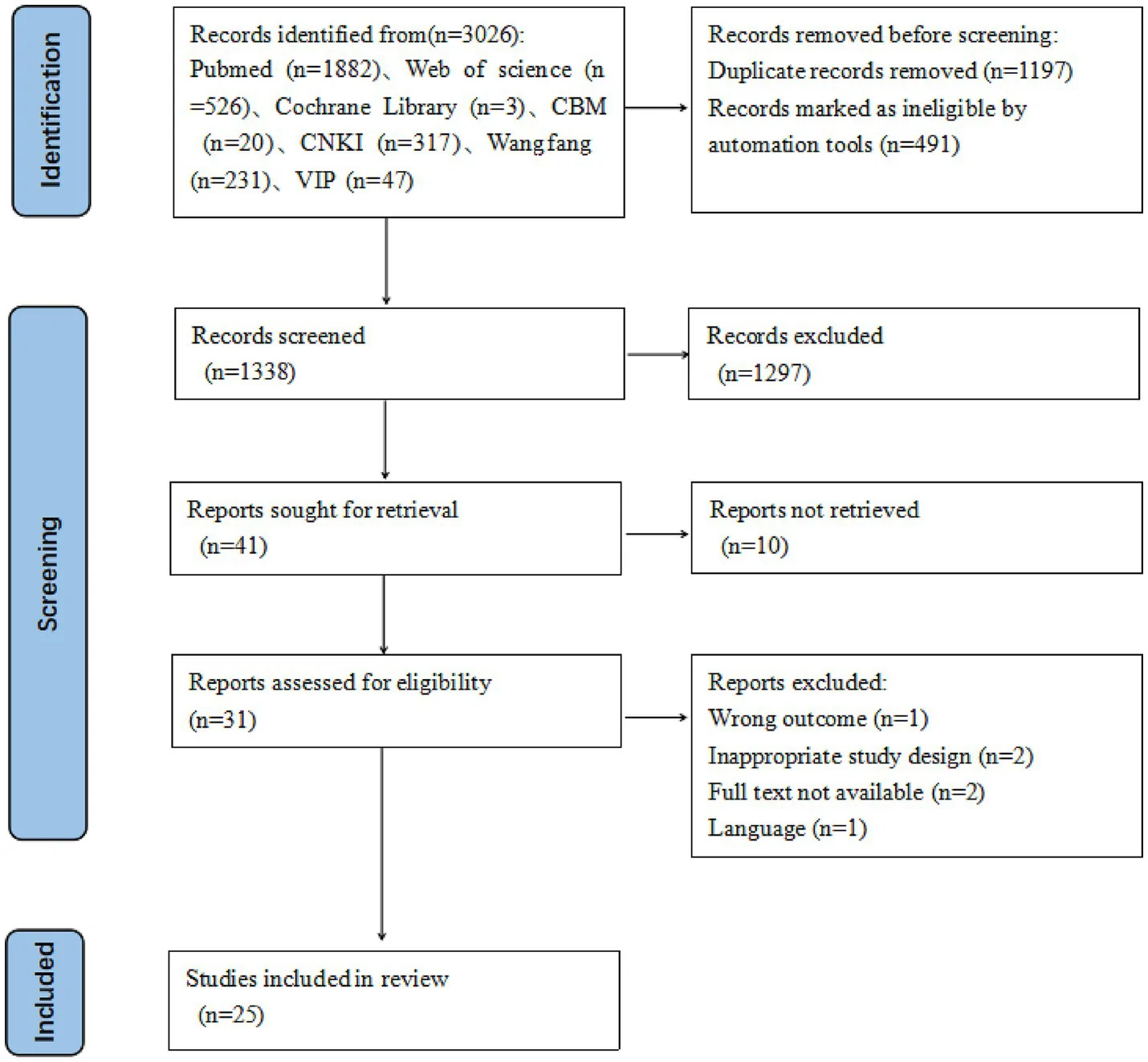
PRISMA flow diagram.

A total of 3,026 records were identified. After removing duplicates and screening titles and abstracts, 31 articles remained. Following full-text review, 25 studies were included (16–40). The PRISMA flow diagram is shown in Figure 1.

### 3.2 Basic characteristics of included studies

This study included a total of 25 studies (16–40), of which 24 studies (16–29, 31–40) were published by Chinese scholars and 1 study (30) was published by Israeli scholars. In terms of publication time, 24 studies were published within the past 5 years. With respect to study populations, all studies involved adult critically ill patients receiving enteral nutrition support, including patients with sepsis, severe stroke, severe acute pancreatitis, neurosurgical critically ill patients, patients receiving prone position ventilation, and patients receiving noninvasive ventilation. Regarding study design, 15 studies (16, 20, 23, 26, 29–33, 35–37, 39, 40) were retrospective cohort studies, 6 studies (17–19, 22, 25, 27) were prospective cohort studies, and 4 studies (21, 24, 28, 38) were case–control studies. Nineteen studies (16–23, 25, 27, 30–34, 36–38, 40) were single-center studies, and 6 studies (24, 26, 28, 29, 35, 39) were multicenter studies. The sample sizes of the included studies ranged from 118 to 1,584 patients. Outcome definitions of enteral feeding intolerance mainly included increased gastric residual volume, vomiting, abdominal distension, diarrhea, aspiration, gastrointestinal bleeding, and interruption of enteral nutrition or insufficient energy intake due to intolerance. The basic characteristics of included studies are shown in Supplementary Table S1.

### 3.3 Variations in EFI definitions and GRV measurement

The definitions of enteral feeding intolerance varied across the included studies. GRV was the most commonly reported objective component, although the reported thresholds and measurement windows differed considerably. Two studies defined EFI using a single GRV ≥ 200 mL measured 4 h after enteral nutrition initiation (22, 30), whereas 1 study used a single GRV > 200 mL within 6 h together with a cumulative GRV ≥ 250 mL within 6 h (24), and another adopted a threshold of GRV > 250 mL after 6 h (36). Other studies used cumulative 24-h thresholds, including GRV ≥ 200 mL/24 h (29), GRV > 250 mL/24 h (28), and GRV ≥ 500 mL/24 h (23, 32). Several studies reported gastric retention or elevated GRV without specifying a quantitative threshold (17–21, 25, 26, 33, 34). In addition to GRV, EFI definitions commonly incorporated vomiting, reflux, diarrhea, abdominal distension, aspiration, gastrointestinal bleeding, decreased or absent bowel sounds, interruption or suspension of enteral nutrition, or failure to achieve target energy intake within 72 h (16, 17, 20, 23, 24, 26–29, 32, 35–40).

Based on the reported components, EFI definitions were classified into four categories: single-time-point GRV definitions; cumulative or 24-h GRV definitions; composite definitions combining GRV with gastrointestinal symptoms and/or inadequate energy intake; and symptom-based definitions without a quantified GRV threshold. Most studies reported sensitivity and specificity for the overall prediction model but did not report performance measures for individual EFI components.

### 3.4 Methods for model construction and predictive performance

Among the 25 included studies (16–40), the incidence of enteral feeding intolerance ranged from 26.25 to 61.3%. All 25 studies used logistic regression, either alone or in combination with other modeling approaches. Five studies (21, 30, 33, 35, 40) employed random forest algorithms, three studies (33, 35, 40) used support vector machines, two studies (21, 30) used gradient boosting trees, one study (21) used deep learning, one study (30) used XGBoost, two studies (30, 33) used k-nearest neighbor algorithms, one study (30) used AdaBoost, two studies (30, 33) used decision trees, and one study (21) used a naive Bayes algorithm.

All 25 studies performed internal validation, and nine studies (21, 23–28, 31, 36) conducted external validation. Seventeen studies (16–20, 22–25, 27, 29, 32, 34, 36–39) used bootstrap resampling, six studies (26, 28, 31, 33, 35, 40) used random split validation, one study (30) used 10-fold cross-validation, and one study (21) used five-fold cross-validation. Twenty-four studies (16–23, 25–40) reported AUC values, which ranged from 0.70 to 0.99. In studies comparing multiple algorithms, comparisons were primarily based on discrimination measures such as AUC. Calibration and external validation were less frequently reported. Detailed information is presented in Supplementary Table S2.

### 3.5 Predictive factors included in the models

All 25 included studies (16–40) reported the presentation format of their prediction models. Twenty studies presented nomograms, ten studies (16, 19, 20, 23, 28, 29, 31, 32, 34, 39) provided regression equations, and five studies (21, 30, 33, 35, 40) presented machine learning models. APACHE II score, age, albumin, blood glucose, mechanical ventilation, and intra-abdominal pressure were the most frequently included predictors. Blood glucose and intra-abdominal pressure were among the most frequently reported continuous predictors. Reporting of measurement timing, modeling form, cut-off values, cut-off selection methods, nonlinear associations, time-dependent changes, and repeated measurements varied across studies. The presentation formats and predictors included in individual models are shown in Table 1.

**Table 1:** Model presentation and predictive factors of risk prediction models.

First author	Model presentation	Predictive factors
Wang et al. (16)	Nomogram + regression equation	Age, blood glucose, timing of enteral nutrition initiation, dietary fiber, intra-abdominal pressure
Gao (17)	Nomogram	Acute gastrointestinal injury (AGI) grade, timing of enteral nutrition initiation, mean enteral nutrition infusion rate, C-reactive protein, albumin, early enema, glutamine supplementation
Li et al. (18)	Nomogram	Length of ICU stay, Glasgow Coma Scale score, blood glucose, serum potassium, hypertension, mechanical ventilation, use of sedative and analgesic medications
Su et al. (19)	Regression equation	APACHE II score, NRS 2002 score, intra-abdominal pressure, albumin
Sun et al. (20)	Nomogram + regression equation	Age, use of two or more antimicrobial agents, probiotics, mechanical ventilation
Hu et al. (21)	Machine learning model	Lower respiratory tract infection, type of enteral nutrition formula, septic shock
Lu et al. (22)	Nomogram	Age, gastrointestinal disease, early feeding, mechanical ventilation, serum sodium
Dong et al. (23)	Regression equation	Triglycerides, hypoproteinemia, intra-abdominal pressure, APACHE II score, timing of enteral nutrition initiation
Liu et al. (24)	Nomogram	Age, APACHE II score, duration of bed rest, albumin, use of vasoactive drugs, head-of-bed elevation angle
Lu et al. (25)	Nomogram	Albumin, presence of gastrointestinal disease, blood glucose, NRS 2002 score, use of acid-suppressive agents
Pan et al. (26)	Nomogram	Mean arterial pressure, Glasgow Coma Scale score, combined use of two or more antibiotics, fluid input and output
Wang et al. (27)	Nomogram	Primary diagnosis, AGI grade, APACHE II score
Zang et al. (28)	Nomogram + regression equation	Triglycerides, hypoproteinemia, APACHE II score, intra-abdominal pressure, timing of enteral nutrition initiation
Zhu et al. (29)	Nomogram + regression equation	APACHE II score, continuous renal replacement therapy (CRRT), intra-abdominal pressure, modified Nutrition Risk in the Critically Ill (mNUTRIC) score, low-calorie energy intake
Raphaeli et al. (30)	Nomogram	Gastric residual volume, APACHE II score, age, Sequential Organ Failure Assessment (SOFA) score, diarrhea, body mass index
Bu et al. (31)	Nomogram + regression equation	Age, APACHE II score, blood glucose, comorbid neurological disease, mechanical ventilation, use of sedative and analgesic agents, use of prokinetic agents
Chen et al. (32)	Nomogram + regression equation	Age, bowel sounds, blood glucose, timing of enteral nutrition initiation, head-of-bed elevation angle, APACHE II score, dietary fiber
Ding et al. (33)	Machine learning model + nomogram	Diuretics, mechanical ventilation, Glasgow Coma Scale score, use of vasoactive drugs, albumin
Liu et al. (34)	Nomogram + regression equation	Blood glucose, intra-abdominal pressure, antibiotic use, serum potassium, feeding volume per administration, albumin, APACHE II score
Wang et al. (35)	Machine learning model	Intra-abdominal pressure, APACHE II score, blood glucose, analgesic use, early enema, mechanical ventilation, use of probiotics
Wang et al. (36)	Nomogram	Blood glucose, gastrin, albumin, gastrin
Wei et al. (37)	Nomogram	Age, length of hospital stay, serum potassium, blood glucose, Glasgow Coma Scale score, mechanical ventilation, use of sedative and analgesic drugs
Yang et al. (38)	Nomogram	SOFA score, NRS 2002 score, analgesic use, length of ICU stay
Yang et al. (39)	Nomogram + regression equation	Daily volume of enteral nutrition formula, sedative use, use of vasoactive drugs, use of prokinetic agents, probiotics, blood glucose, lactate
Yu et al. (40)	Machine learning model	Blood glucose, mechanical ventilation, use of vasoactive drugs, albumin, serum potassium

APACHE II, Acute Physiology and Chronic Health Evaluation-II, NRS2002, Nutrition Risk Screening 2002, AGI, Acute Gastrointestinal Injury, GCS, Glasgow Coma Scale, mNutric, modified nutrition risk in the critically ill, CRRT, continuous renal replacement therapy, SOFA, Sequential Organ Failure Assessment.

### 3.6 The methodological quality assessment of included studies

#### 3.6.1 Study population domain

Among the included studies, 15 studies (16, 20, 23, 26, 29–33, 35–37, 39, 40) were retrospective cohort studies, and 19 studies (16–23, 25, 27, 30–34, 36–38, 40) were single-center studies. These studies were judged to have a high risk of bias.

#### 3.6.2 Predictor domain

All 25 included studies (16–40) used consistent definitions and assessment methods for predictors across study populations and clearly specified evaluation criteria. Most included studies clearly described predictor definitions and measurement methods, and predictors were generally based on routinely available clinical variables. Therefore, the predictor domain was considered to have relatively low applicability concerns.

#### 3.6.3 Outcome domain

All 25 studies (16–40) were rated as having a relatively high risk of bias in the outcome domain. The operational definitions of EFI varied across studies. Reported outcome components included different GRV thresholds, vomiting, reflux, diarrhea, abdominal distension, aspiration, gastrointestinal bleeding, bowel sound changes, interruption or suspension of enteral nutrition, and failure to achieve target energy intake.

#### 3.6.4 Statistical analysis domain

All 25 studies (16–40) were rated as having a high risk of bias in the statistical analysis domain. Six studies (16, 18, 21, 24, 26, 34) had insufficient sample sizes during model development or validation. Nineteen studies (16–23, 25, 27, 30–34, 36–38, 40) were single-center studies, six studies (26, 28, 31, 33, 35, 40) used random split validation, three studies (30, 35, 40) did not report calibration, nine studies (21, 23–28, 31, 36) conducted external validation, and four studies (16, 21, 22, 29) did not clearly report methods for handling missing data.

#### 3.6.5 Applicability assessment

Applicability was assessed across the participant, predictor, and outcome domains. All 25 studies (16–40) were rated as having low concerns regarding applicability in these three domains according to PROBAST. Detailed assessments of risk of bias and applicability are presented in Supplementary Table S3.

### 3.7 Clinical impact and intervention effects of prediction models

None of the included studies directly evaluated the effects of interventions guided by the developed prediction models. Although the studies reported model performance indicators such as AUC, sensitivity, specificity, calibration, and validation results, no study assessed whether applying the model in clinical practice improved enteral nutrition delivery or patient outcomes. Specifically, there was no evidence from the included studies that model-guided management reduced the incidence of enteral feeding intolerance, improved target energy achievement, reduced feeding interruption, aspiration, infection, ICU length of stay, or mortality.

## 4 Discussion

### 4.1 Frequently reported predictors and heterogeneity across models

The results of this review showed substantial heterogeneity in both candidate predictor selection and final predictor retention across the included models. No single predictor was consistently identified across all included studies. APACHE II score, blood glucose, mechanical ventilation, age, albumin, and intra-abdominal pressure were relatively frequently reported predictors, but each appeared only in a subset of models. Therefore, these predictors should be interpreted as recurrent signals or hypothesis-generating factors rather than definitive or universally applicable predictors of enteral feeding intolerance. Differences in study populations, EFI definitions, candidate predictor sets, variable selection methods, and validation strategies may all have contributed to the variability in predictors retained across models.

Eleven studies (19, 23, 24, 27–32, 34, 35) identified APACHE II score as a predictor of enteral feeding intolerance. APACHE II is a global measure of physiological derangement and disease severity rather than a gastrointestinal-specific measure. Reviews of gastrointestinal dysmotility and feed intolerance in critically ill patients have described impaired splanchnic perfusion, systemic inflammation, autonomic and hormonal disturbances, organ dysfunction, and treatment-related factors as potential mechanisms linking greater illness severity to impaired gastrointestinal function (41, 42). Thus, a high APACHE II score may identify patients with a greater overall burden of physiological dysfunction and a higher probability of EFI, but it should not be interpreted as a direct cause of feeding intolerance. Clinically, APACHE II may contribute to initial risk stratification, but its predictive value should be considered together with gastrointestinal symptoms, hemodynamic status, medication exposure, and other patient-specific factors.

Eleven studies (16, 18, 25, 31, 32, 34–37, 39, 40) identified blood glucose as a predictor. Experimental evidence indicates that acute hyperglycemia can slow gastric emptying, including at glucose concentrations within the postprandial range (43). Diabetes and persistent dysglycemia may additionally affect gastrointestinal motility through autonomic, neural, hormonal, and smooth-muscle mechanisms (44). These findings provide biological plausibility for blood glucose as a potential predictor of EFI. Nevertheless, the available evidence does not establish a universally applicable glucose threshold for predicting EFI in critically ill patients. Moreover, a single glucose measurement may not adequately represent glycemic variability during critical illness. Future studies should therefore report measurement timing, assess repeated or time-updated glucose values, and evaluate possible nonlinear associations rather than relying solely on study-specific cut-off points.

Eight studies (18, 20, 22, 31, 33, 35, 37, 40) retained mechanical ventilation as a predictor of enteral feeding intolerance. However, the original studies generally used the broad term “mechanical ventilation” and did not consistently distinguish invasive mechanical ventilation from non-invasive ventilation. Heyland et al. showed that EFI was common and clinically relevant in a large cohort of mechanically ventilated critically ill patients (45). However, because all patients in that analysis were mechanically ventilated, the study did not establish mechanical ventilation itself as an independent causal factor or determine whether the association differed by ventilation modality. Mechanical ventilation may also act as a proxy for illness severity and treatment intensity, including sedation, opioid administration, vasopressor use, immobility, and positive-pressure-related changes in abdominal physiology. Accordingly, this predictor should be interpreted cautiously. Future studies should clearly specify ventilation modality, airway interface, ventilatory parameters, duration of ventilation, and timing relative to enteral nutrition initiation.

Eight studies (16, 19, 22, 24, 30–32, 37) identified age as a predictor. Liu et al. (24) reported a higher risk of EFI among older patients. Although aging is associated with changes in gastrointestinal motility, sensory function, mucosal physiology, and the prevalence of gastrointestinal disorders, aging itself appears to have only a modest direct effect on many gastrointestinal functions (46). The observed predictive value of age may therefore also reflect frailty, comorbidity burden, polypharmacy, neurological disease, reduced mobility, and greater illness severity. Advanced age may be useful as part of a multivariable risk assessment, but it should not be used as a standalone criterion for restricting or interrupting enteral nutrition.

Eight studies (17, 19, 24, 25, 33, 34, 36, 40) identified albumin as a predictor. This finding requires particularly cautious interpretation. Serum albumin is influenced by systemic inflammation, hepatic protein reprioritization, capillary permeability, fluid balance, and disease severity. The ASPEN position paper states that albumin and prealbumin characterize inflammatory burden rather than nutritional intake or protein-energy malnutrition and should not be used as direct nutritional markers (47). Therefore, the association between low albumin and EFI does not demonstrate that hypoalbuminemia directly causes feeding intolerance. Albumin may instead function as a proxy for inflammatory burden, capillary leakage, fluid overload, or overall physiological severity. It should be interpreted alongside hemodynamic status, fluid balance, gastrointestinal function, disease severity, and actual nutritional delivery.

Seven studies (16, 19, 23, 28, 29, 34, 35) identified intra-abdominal pressure as a predictor. The World Society of the Abdominal Compartment Syndrome defines intra-abdominal hypertension as a sustained or repeated pathological elevation in intra-abdominal pressure and recommends standardized measurement in at-risk patients (48). Elevated intra-abdominal pressure can impair abdominal organ perfusion, alter abdominal compliance, and contribute to bowel-wall edema and organ dysfunction (48, 49). These mechanisms provide biological plausibility for an association with impaired gastrointestinal function and EFI. Nevertheless, the available studies do not establish a single EFI-specific intra-abdominal pressure threshold, and measurement technique, body position, ventilation, fluid balance, and measurement timing can substantially affect recorded values. Intra-abdominal pressure should therefore be considered as one component of a broader gastrointestinal and hemodynamic assessment rather than an isolated trigger for feeding interruption.

Overall, the predictors discussed above should not be regarded as definitive causal determinants of EFI. Instead, they represent variables that were relatively frequently retained in existing prediction models. The heterogeneity in retained predictors suggests that current evidence remains insufficient to support a single universal predictor set for critically ill patients. Future studies should use standardized EFI definitions, prespecified candidate predictor sets, transparent variable selection strategies, and external validation to determine whether these predictors have stable and generalizable predictive value across different clinical settings.

### 4.2 Handling of threshold effects in continuous and dynamic predictors

Several frequently reported predictors, particularly blood glucose and intra-abdominal pressure, are continuous variables that can change substantially during critical illness and enteral nutrition. Their associations with EFI may therefore be nonlinear and time dependent. Acute hyperglycemia can influence gastric emptying (43), whereas sustained intra-abdominal hypertension can affect abdominal perfusion and organ function (48, 49). However, these observations do not imply that every small increase carries the same clinical significance or that a universal threshold can be applied across populations.

The original studies addressed continuous predictors inconsistently. Some included blood glucose or intra-abdominal pressure on their original continuous scales, whereas others converted them into categories or incorporated cut-off points into nomograms. Dichotomizing continuous predictors can discard prognostic information, reduce statistical power, impose an artificial step change in risk, and produce cut-off points that are unstable across datasets (50). When thresholds were reported, they were commonly derived from study-specific ROC analyses, univariable screening, or clinical convention rather than prespecified and independently validated criteria.

Most studies also relied on a single measurement obtained before or near the initiation of enteral nutrition. Few considered repeated measurements, time-updated values, cumulative exposure, or temporal trends. Methods such as restricted cubic splines, fractional polynomials, generalized additive models, landmark approaches, and joint or dynamic prediction models can be used to examine nonlinear and time-dependent predictor effects (50, 51), but these approaches were rarely reported in the included studies.

Inconsistent handling of continuous and dynamic predictors may contribute to between-study heterogeneity. Differences in measurement timing, categorization, cut-off selection, and functional form can alter estimated predictor effects, calibration, and model transportability (50–52). Predictor–outcome overlap should also be assessed. For example, when intra-abdominal pressure-related findings or gastrointestinal dysfunction measures contribute both to predictor assessment and outcome classification, apparent model performance may be inflated.

Future studies should retain continuous variables on their original scales whenever appropriate, assess plausible nonlinear relationships, prespecify measurement windows, and report repeated or time-updated measurements where clinically relevant. Any proposed cut-off point should be justified clinically and statistically and validated in an independent population before being used to guide enteral nutrition management.

### 4.3 Modeling approaches, validation strategies, calibration, and clinical interpretability

The results of this review showed that the included prediction models generally reported acceptable to excellent discrimination, with AUC values ranging from 0.70 to 0.99. However, high discrimination alone does not necessarily indicate that a model is methodologically robust or clinically reliable. Among the 25 included studies (16–40), logistic regression was the most frequently used modeling approach, whereas 5 studies (21, 30, 33, 35, 40) applied one or more machine-learning algorithms. Logistic regression remains widely used because its structure is comparatively transparent and can be presented as a regression equation, risk score, or nomogram. Machine-learning methods may accommodate complex nonlinear relationships and interactions, but such flexibility does not guarantee superior performance. A systematic review comparing machine-learning methods with logistic regression found no evidence of superior performance when comparisons at low risk of bias were considered (53). Consistent with that evidence, the studies included in the present review did not demonstrate a clear and generalizable advantage of machine learning over logistic regression. Reported high AUC values were frequently based on limited samples, internal validation, or random data splitting and were not consistently accompanied by calibration assessment or independent external validation.

External validation remains insufficient in the current evidence base. Nineteen studies (16–23, 25, 27, 30–34, 36–38, 40) were single-center studies, whereas only 6 studies (24, 26, 28, 29, 35, 39) were multicenter studies. Models developed in a single institution may capture local case mix, measurement practices, feeding protocols, and clinical decision patterns, thereby limiting transportability (52). Only 9 studies (21, 23–28, 31, 36) conducted external validation. Previous methodological reviews have shown that independent external validation is uncommon and that model performance, particularly discrimination, often decreases when models are evaluated in new populations (54, 55). Therefore, existing EFI models should undergo multicenter external validation and, where necessary, recalibration or model updating before additional new models are developed.

The choice of internal validation strategy may also influence the reported predictive performance, especially when sample sizes are limited. All 25 studies (16–40) performed internal validation, but only nine studies (21, 23–28, 31, 36) conducted external validation. With respect to internal validation methods, 17 studies (16–20, 22–25, 27, 29, 32, 34, 36–39) used bootstrap resampling, six studies (26, 28, 31, 33, 35, 40) used random split validation, one study (30) used 10-fold cross-validation, and one study (21) used five-fold cross-validation. Bootstrap validation makes efficient use of the full dataset and can estimate optimism-corrected performance, which may be particularly useful for small or moderate development samples. In contrast, random split validation is simple and intuitive, but it reduces the effective sample size available for both model development and validation. This approach may produce unstable estimates when the number of outcome events is limited. Cross-validation can improve data efficiency compared with a single random split, but its reliability depends on adequate event numbers within each fold and appropriate model tuning. Therefore, differences in internal validation strategies across the included studies may partly explain the heterogeneity in reported model performance. Future studies should select validation methods according to sample size, number of outcome events, and model complexity, and should report optimism-corrected performance whenever possible (52, 56, 57).

Calibration is another critical issue. AUC mainly reflects discrimination, namely the ability of a model to distinguish between patients who will and will not develop enteral feeding intolerance. However, AUC does not indicate whether the predicted probabilities are accurate. A poorly calibrated model may overestimate or underestimate the absolute risk of feeding intolerance even when the AUC is high (58). This has important clinical safety implications. If a model overestimates risk, patients may receive unnecessary interventions, such as excessive feeding interruption, inappropriate prokinetic therapy, or premature post-pyloric feeding. Conversely, if a model underestimates risk, high-risk patients may not receive timely preventive measures. In the included studies, three studies (30, 35, 40) did not report calibration. This limits the clinical reliability and safety of applying these models in practice. Future studies should report calibration plots, calibration intercepts and slopes, Brier scores, and decision-curve analysis in addition to AUC, so that both statistical performance and clinical usefulness can be evaluated (58).

The trade-off between predictive accuracy and clinical interpretability should also be considered. Nomogram and regression-based models are relatively transparent and easy to use in clinical practice, allowing clinicians to understand how individual predictors contribute to risk. This is particularly important for enteral nutrition management, because clinicians need to understand why a patient is classified as high risk before adjusting feeding rate, monitoring intensity, or preventive interventions. In contrast, complex machine learning models used in some studies (21, 30, 33, 35, 40) may achieve higher apparent accuracy in specific datasets, but they are often less interpretable and may be more difficult to reproduce, calibrate, and implement at the bedside. Therefore, future model development should not focus solely on maximizing AUC. Instead, model evaluation should balance discrimination, calibration, external validation, clinical interpretability, ease of use, and clinical impact (53, 55, 58).

In addition, several methodological limitations should be noted. Six studies (16, 18, 21, 24, 26, 34) had insufficient sample sizes during model development or validation. Adequate sample size is essential for developing stable prediction models, and sample size estimation should consider the number of candidate predictors and outcome events. The events-per-variable principle remains an important reference for reducing overfitting and improving model stability (56, 57). Four studies (16, 21, 22, 29) did not clearly describe methods for handling missing data. Complete-case analysis is convenient, but it can lead to loss of information and biased estimates when missingness is substantial, whereas appropriate imputation methods may help preserve sample size and reduce bias (59).

### 4.4 Heterogeneity in EFI definitions, outcome misclassification, and the role of GRV

The definition of enteral feeding intolerance varied substantially across the included studies (16–40). Differences involved GRV thresholds, measurement timing and frequency, gastrointestinal symptoms, feeding interruption, and failure to achieve energy targets. This finding is consistent with previous reviews showing that EFI lacks a universally accepted definition and has been operationalized using numerous combinations of GRV, gastrointestinal symptoms, and inadequate enteral nutrition delivery (60–63).

GRV was the most frequently reported objective component, but assessment methods were inconsistent. Some studies used a single post-feeding value, whereas others used short-term or 24-h cumulative volumes. Several studies described gastric retention or elevated GRV without reporting a quantitative threshold. Other definitions relied on vomiting, reflux, diarrhea, abdominal distension, aspiration, gastrointestinal bleeding, reduced bowel sounds, feeding interruption, or failure to achieve target energy intake. The clinical value of routine GRV monitoring and the optimal GRV threshold remain uncertain, and randomized and systematic-review evidence has not established that GRV monitoring consistently improves major clinical outcomes (8, 64).

Most original studies reported sensitivity, specificity, discrimination, and other performance measures only for the overall EFI outcome rather than separately for individual outcome components. Therefore, this review could not perform a formal component-level stratified analysis according to specific GRV thresholds, measurement windows, or symptom patterns. Consequently, the present findings do not allow conclusions regarding whether a particular GRV threshold, measurement approach, or symptom component provides superior sensitivity, specificity, or predictive value. Differences in GRV assessment and EFI components should therefore be interpreted as an important source of between-study heterogeneity rather than evidence of differential subgroup effects. The lack of component-level reporting also limits the ability to determine whether variation in model performance was attributable to EFI definitions or to differences in study populations, predictor selection, modeling methods, and validation strategies (61–64).

Heterogeneous EFI definitions may also introduce outcome misclassification. In studies where EFI was defined using GRV thresholds, feeding interruption, or failure to achieve target energy intake, outcome classification may partly reflect local monitoring protocols and clinical feeding decisions rather than physiological gastrointestinal intolerance alone (61). For example, an elevated GRV measurement may prompt clinicians to reduce or suspend enteral feeding, after which inadequate nutrition delivery may subsequently be classified as part of the EFI outcome. In this situation, failure to achieve target energy intake may represent a downstream consequence of GRV-based management rather than an independent manifestation of gastrointestinal intolerance (58). Therefore, outcome assessment may be influenced by institutional practices, including the frequency of GRV monitoring, thresholds for feeding interruption, and local enteral nutrition protocols (61, 63, 64).

This issue has important implications for prediction-model development and interpretation. When outcome classification is partly determined by clinical management decisions or local monitoring practices, prediction models may capture institution-specific care patterns in addition to the underlying physiological risk of feeding intolerance. This may contribute to apparently favorable model performance in the development setting while reducing transportability to institutions that use different GRV thresholds, monitoring schedules, feeding protocols, or criteria for interrupting enteral nutrition (61–64). These concerns were reflected in the PROBAST assessment, in which the outcome domain was considered to have a relatively high risk of bias because standardized EFI criteria were lacking and outcome misclassification could not be excluded.

Future studies should adopt prespecified and standardized EFI definitions and clearly report each component used to determine the outcome. In particular, studies should report the GRV threshold, measurement timing, measurement frequency, whether GRV represents a single value or cumulative volume, the gastrointestinal symptoms included, and the criteria used for feeding interruption or inadequate energy delivery. When composite EFI outcomes are used, the occurrence and model performance of individual components should be reported separately where possible. Such reporting would facilitate component-level comparisons, improve consistency in outcome assessment, and enhance the comparability, external validation, and clinical transportability of EFI prediction models.

### 4.5 Clinical utility and intervention impact of prediction models

Although the included models showed acceptable to excellent discrimination, evidence regarding their clinical utility remains limited. Prediction model performance, such as AUC, sensitivity, specificity, and calibration, reflects statistical accuracy, but does not necessarily indicate that using the model will improve clinical decision-making or patient outcomes (58). In this review, the included studies mainly focused on model development and validation, and no study formally evaluated whether model-guided interventions improved enteral nutrition management or clinical outcomes.

The purpose of EFI prediction should be to support assessment and appropriately targeted care rather than to automatically interrupt feeding. Depending on the clinical context, a high predicted risk might prompt closer evaluation of gastrointestinal symptoms, review of sedative and opioid exposure, correction of reversible contributors, cautious feeding advancement, selected use of prokinetic therapy, or consideration of post-pyloric feeding. Current critical-care nutrition guidelines support individualized management and emphasize avoiding unnecessary interruption of enteral nutrition rather than using a single risk factor or GRV value in isolation (4, 65, 66). However, these guideline-supported measures have not yet been shown to improve outcomes when triggered specifically by the EFI models reviewed here.

Prospective impact studies are therefore required. Prediction-model impact can be evaluated using comparative implementation studies, before-and-after designs, cluster-randomized trials, or individually randomized trials, provided that the model has first demonstrated adequate external validity and usability (67). Decision-curve analysis may estimate the net benefit of using a model across clinically relevant risk thresholds, but it cannot replace prospective evidence that model-guided care changes decisions or improves patient outcomes (68).

Future impact studies should assess target energy achievement, unnecessary feeding interruption, vomiting, aspiration, infection, duration of mechanical ventilation, ICU length of stay, mortality, and treatment-related harms. They should also examine whether false-positive risk classification causes excessive monitoring, prokinetic exposure, post-pyloric tube placement, or avoidable feeding restriction. Only after such evaluation can the effectiveness and safety of these models as clinical decision-support tools be established.

### 4.6 Implications for future research

Based on the results of this systematic review, future research on risk prediction models for enteral feeding intolerance in critically ill patients needs further standardization in terms of outcome definitions, study design, and clinical translation of models. First, there is currently no unified standard for the definition of feeding intolerance with respect to gastric residual volume thresholds, combinations of gastrointestinal symptoms, and whether insufficient energy intake should be included. Such heterogeneity may affect the comparability of study results and the external applicability of prediction models. Future studies should promote the standardization of outcome measures based on evidence-based data (61–64). Second, research should shift from repeated single-center model development toward multicenter prospective validation. Existing models should be evaluated across different ICU types, disease groups, healthcare systems, and nutrition protocols. External validation should include discrimination, calibration, overall performance, and assessment of whether recalibration or model updating is required (52, 54, 55). Third, model-development procedures should be prespecified and methodologically appropriate. Candidate predictors should be selected according to clinical availability and biological plausibility rather than univariable statistical significance alone. Sample size should be calculated according to the number of predictor parameters, outcome prevalence, and anticipated model performance (57). Continuous predictors should not be dichotomized without strong justification, missing data should be handled appropriately, and predictor measurement timing should be clearly defined. Studies should follow contemporary guidance for the development and reporting of clinical prediction models (69, 70). Fourth, machine learning should not be adopted solely because it produces a higher apparent AUC in one dataset. Comparisons between machine-learning and regression models should use the same predictor information, data partitions, tuning procedures, and validation framework. Model stability should be assessed because small or sparse datasets can yield substantially different predictor effects and individual risk estimates when the analysis is repeated (53, 71). Interpretability, calibration, reproducibility, and external validity should be considered alongside discrimination. Finally, models that demonstrate adequate external performance should proceed to implementation and impact evaluation. Decision-curve analysis may help identify plausible risk thresholds, but prospective comparative studies remain necessary to determine whether model use improves care and patient outcomes (67, 68).

### 4.7 Limitations

This review has several limitations. First, this study included only Chinese- and English-language publications, which may have led to the omission of relevant studies published in other languages. In addition, most included studies were conducted in China, which may limit the generalizability of the findings to other healthcare systems, ethnic populations, nutrition protocols, and ICU settings. Regional differences in patient characteristics, feeding practices, GRV monitoring, and thresholds for interrupting enteral nutrition should therefore be considered when interpreting the findings.

Second, the included prediction models had important methodological limitations. Most studies were retrospective or single-center, and external validation was insufficient. These limitations may reduce model robustness and transportability. Because few studies directly compared traditional regression and machine learning models under the same validation framework, and because calibration and external validation were inconsistently reported, this review could not determine whether machine learning models offer a robust clinical advantage over regression-based models.

Third, the lack of a standardized definition of enteral feeding intolerance across studies represents a major limitation. The included studies used heterogeneous EFI definitions, especially regarding GRV thresholds, measurement timing, gastrointestinal symptom combinations, feeding interruption, and failure to achieve target energy intake. Several studies defined EFI using GRV thresholds, feeding interruption, or inadequate nutrition delivery, which may partly reflect local monitoring practices and clinical feeding decisions rather than true physiological gastrointestinal intolerance. Therefore, outcome misclassification may have occurred. Such misclassification could influence predictor selection, apparent model performance, calibration, and between-study heterogeneity.

Fourth, because most studies reported model performance only for the overall EFI outcome and did not separately report performance for individual outcome components, this review was unable to conduct formal stratified analyses according to GRV thresholds, measurement windows, or symptom patterns. Consequently, we could not determine whether the observed differences in model performance were attributable to specific EFI definitions or to other differences in study populations, predictor selection, and modeling methods. This limitation should be considered when interpreting the between-study heterogeneity and generalizability of the findings.

Fifth, all included studies used observational designs, including retrospective or prospective cohort studies and case–control studies. Although prediction modeling does not aim to estimate causal effects in the same way as etiological studies, residual confounding and unmeasured clinical factors may still influence which predictors are selected and how their associations are interpreted. For example, variables such as albumin, mechanical ventilation, vasopressor use, sedation, glycemic control, fluid balance, and disease severity may be interrelated. Some predictors retained in the models may therefore act as proxy markers of overall illness severity, inflammation, treatment intensity, or local clinical practice rather than independent biological determinants of EFI. Because individual studies varied in candidate predictor selection, variable selection procedures, and adjustment for clinically important factors, the identified predictors should be interpreted as hypothesis-generating rather than causal or universally applicable.

Finally, the included studies did not provide direct evidence on the effectiveness of model-guided interventions. Therefore, this review could evaluate the statistical performance of existing models, but could not determine whether their use improves enteral nutrition management or patient outcomes in clinical practice. Prospective implementation studies or randomized trials are needed to assess whether model-guided feeding protocols can improve target energy achievement, reduce feeding interruption, prevent aspiration or infection, shorten ICU stay, or improve survival.

## 5 Conclusion

This systematic review comprehensively evaluated risk prediction models for enteral feeding intolerance in critically ill patients. Existing models, largely based on routine clinical indicators, demonstrated acceptable to excellent discrimination; however, their methodological quality varied considerably, and their clinical applicability remains uncertain. APACHE II score, blood glucose, mechanical ventilation, age, albumin, and intra-abdominal pressure were relatively frequently reported predictors, but no single predictor was consistently identified across all models. Therefore, these factors should be interpreted as recurrent signals rather than definitive or universally applicable predictors. Most included studies were retrospective and single-center, with limited external validation and methodological shortcomings in sample size estimation, missing data handling, calibration assessment, and outcome definition. The heterogeneous definitions of enteral feeding intolerance, particularly differences in GRV thresholds, symptom combinations, feeding interruption, and failure to achieve target energy intake, may contribute to outcome misclassification and between-study heterogeneity. In addition, current evidence mainly focuses on model development and validation, while direct evidence on model-guided clinical interventions and patient-relevant outcomes remains lacking. Future studies should adopt standardized and clinically meaningful EFI definitions, conduct multicenter prospective research, prespecify candidate predictors, standardize model development and validation procedures, strengthen calibration and external validation, and evaluate the clinical impact of model-guided enteral nutrition management. These steps are necessary before existing prediction models can be confidently applied as clinical decision-support tools in critically ill patients.

## Data Availability

The original contributions presented in the study are included in the article/Supplementary material, further inquiries can be directed to the corresponding author.
