# Supplementary material for: Risk prediction models for enteral feeding intolerance in critically ill patients: a systematic review
Source: Front Public Health. 2026 Sep 9;14:1900308. doi: 10.3389/fpubh.2026.1900308 (PMC13597850; doi:10.3389/fpubh.2026.1900308)
Supplement: Supplementary file 1 [file Table_1.docx]

**Supplementary Table S1. Basic Characteristics of Included Studies**

| Study | Year | Country | Study Site | Study Population | Study Design | Outcome Definition of Enteral Feeding Intolerance | Sample Size |
| --- | --- | --- | --- | --- | --- | --- | --- |
| Wang et al.[17] | 2019 | China | Shandong Provincial Hospital Affiliated to Shandong University | Patients with severe acute pancreatitis | Retrospective cohort study | Gastrointestinal adverse reactions (abdominal distension, diarrhea, constipation) occurring during enteral nutrition leading to suspension or termination of enteral nutrition, resulting in failure to reach the target energy intake of 83.68 kJ/(kg·d) within 72 h | 118 |
| Gao[18] | 2021 | China | The First Affiliated Hospital of Anhui Medical University | Adult ICU patients with sepsis | Prospective cohort study | Occurrence of vomiting and/or reflux, diarrhea, decreased or absent bowel sounds, constipation, gastric retention, gastrointestinal bleeding, or failure to achieve the target energy requirement (20 kcal/kg·d) within 72 h during enteral nutrition, or intolerance caused by clinical reasons (excluding medical or nursing procedures or examinations) | 271 |
| Li et al.[19] | 2022 | China | Shanghai First People’s Hospital | Neurosurgical ICU patients | Prospective cohort study | Occurrence of vomiting, gastric retention, diarrhea, gastrointestinal bleeding, or enterocutaneous fistula | 127 |
| Su et al.[20] | 2022 | China | Changzhou First People’s Hospital | Critically ill patients | Prospective cohort study | Occurrence of vomiting, gastric retention, diarrhea, gastrointestinal bleeding, enterocutaneous fistula, or decreased/absent bowel sounds | 230 |
| Sun et al.[21] | 2022 | China | First Affiliated Hospital of Naval Medical University | Patients with severe stroke | Retrospective cohort study | Four symptoms: vomiting, diarrhea, gastric retention, aspiration. No symptoms = good tolerance; ≥1 symptom but manageable after adjustment = poor tolerance; ≥1 symptom not relieved after intervention and unable to continue enteral nutrition = complete intolerance | 282 |
| Hu et al.[22] | 2022 | China | People’s Hospital of Guangxi Zhuang Autonomous Region | Adult ICU patients with sepsis | Case–control study | Occurrence of gastrointestinal symptoms during enteral nutrition, including vomiting, abdominal distension, high gastric residual volume, diarrhea, or elevated intra-abdominal pressure | 195 |
| Lu et al.[23] | 2022 | China | Shanghai General Hospital, Shanghai Jiao Tong University | Adult ICU patients | Prospective cohort study | Gastric residual volume ≥200 mL after 4 h of enteral nutrition; occurrence of diarrhea, abdominal distension, constipation, vomiting/reflux, or aspiration | 203 |
| Dong et al.[24] | 2023 | China | Affiliated Hospital of Changchun University of Chinese Medicine | Patients with severe acute pancreatitis | Retrospective cohort study | Occurrence of vomiting/reflux, abdominal distension, diarrhea, gastrointestinal bleeding, decreased/absent bowel sounds, constipation, or gastric residual volume ≥500 mL/24 h leading to interruption or suspension of enteral nutrition; failure to reach target energy intake of 83.68 kJ/(kg·d) within 72 h; suspension due to other clinical reasons | 338 |
| Liu et al.[25] | 2023 | China | Three tertiary hospitals in Shandong Province | Patients with severe stroke | Case–control study | Single gastric residual volume >200 mL within 6 h; cumulative gastric residual volume ≥250 mL within 6 h; vomiting, reflux, aspiration, diarrhea, abdominal distension; enteral nutrition intake <83.68 kJ/(kg·d) within 72 h | 218 |
| Lu et al.[26] | 2023 | China | Affiliated Central Hospital of Huzhou University | Critically ill ICU patients | Prospective cohort study | Occurrence of gastrointestinal adverse reactions (diarrhea, constipation, high gastric residual volume, vomiting, gastrointestinal bleeding, abdominal distension); suspension or termination of enteral nutrition for any clinical reason | 451 |
| Pan et al.[27] | 2023 | China | A tertiary hospital in Shandong Province | Neurosurgical critically ill patients | Retrospective cohort study | Gastrointestinal symptoms (diarrhea, abdominal distension, vomiting, reflux, excessive gastric residual volume) causing interruption or suspension of enteral nutrition; energy intake <83.72 kJ/(kg·d) within 72 h | 205 |
| Wang et al.[28] | 2023 | China | First Hospital of Jilin University | Critically ill ICU patients | Prospective cohort study | Intolerance to enteral feeding for any clinical reason, including vomiting, high gastric residual volume (single GRV ≥200 mL), diarrhea (≥3 episodes/day or stool weight >200–250 g/day), gastrointestinal bleeding, enterocutaneous fistula; failure to reach ≥20 kcal/(kg·d) within 72 h | 771 |
| Zang et al.[29] | 2023 | China | A tertiary hospital in Jilin Province | Patients with severe acute pancreatitis | Case–control study | Gastric residual volume >250 mL/24 h; diarrhea, abdominal distension, constipation, vomiting/reflux, decreased/absent bowel sounds, gastrointestinal bleeding; enteral nutrition <83.6 kJ/(kg·d) within 72 h | 351 |
| Zhu et al.[30] | 2023 | China | Zhejiang Hospital research database | ICU patients with sepsis | Retrospective cohort study | Gastric residual volume ≥200 mL/24 h; diarrhea, abdominal distension, intra-abdominal pressure ≥12 mmHg, vomiting/reflux, aspiration | 140 |
| Raphaeli et al.[31] | 2023 | Israel | Rabin Medical Center (Beilinson Hospital) | Adult critically ill ICU patients | Retrospective cohort study | Gastric residual volume ≥200 mL after 4 h of enteral nutrition; diarrhea, abdominal distension, constipation, vomiting/reflux, aspiration | 1584 |
| Pu et al.[32] | 2024 | China | Jinling Hospital, Nanjing University Medical School | Adult ICU patients | Retrospective cohort study | Diarrhea defined as Hart diarrhea score ≥12 within 24 h; abdominal distension reported by patients or confirmed by physical examination | 670 |
| Chen et al.[33] | 2024 | China | Suqian Hospital of Nanjing Drum Tower Hospital Group | Adult ICU patients | Retrospective cohort study | Vomiting, abdominal distension, diarrhea, gastric residual volume ≥500 mL/24 h; energy intake <83.72 kJ/(kg·d) within 72 h | 160 |
| Ding et al.[34] | 2024 | China | Northern Jiangsu People’s Hospital, Yangzhou University | Patients with severe intracerebral hemorrhage | Retrospective cohort study | Gastrointestinal symptoms (diarrhea, constipation, abdominal distension, vomiting, gastrointestinal bleeding, reflux, gastric retention) causing interruption or suspension of enteral nutrition | 485 |
| Liu et al.[35] | 2024 | China | Kunshan Second People’s Hospital | ICU patients receiving prone ventilation | Case–control study | Gastrointestinal symptoms causing interruption or suspension of enteral nutrition | 200 |
| Wang et al.[36] | 2024 | China | A tertiary hospital in Zhejiang Province | Adult ICU patients | Retrospective cohort study | Gastrointestinal symptoms leading to interruption or suspension of enteral nutrition; energy intake <83.72 kJ/(kg·d) within 72 h | 487 |
| Wang et al.[37] | 2024 | China | Hai’an Hospital Affiliated to Nantong University | ICU patients receiving noninvasive ventilation | Retrospective cohort study | Aspiration, gastric retention, diarrhea, vomiting; gastric residual volume >250 mL after 6 h; failure to reach target energy intake after 2 h of enteral nutrition | 258 |
| Wei et al.[38] | 2024 | China | Handan Central Hospital | Patients with severe traumatic brain injury | Retrospective cohort study | Gastrointestinal symptoms causing interruption or suspension of enteral nutrition | 170 |
| Yang et al.[39] | 2024 | China | First Affiliated Hospital of Xinjiang Medical University | ICU patients with sepsis | Case–control study | Energy intake <20 kJ/(kg·d) within 72 h; gastrointestinal symptoms leading to interruption or suspension of enteral nutrition | 199 |
| Yang et al.[40] | 2024 | China | Two tertiary hospitals in Nanchong City | ICU patients with sepsis | Retrospective cohort study | Gastrointestinal symptoms causing interruption or suspension of enteral nutrition | 307 |
| Yu et al.[41] | 2025 | China | Affiliated Hospital of Shaoxing University | Neurosurgical critically ill patients | Retrospective cohort study | A series of gastrointestinal symptoms (vomiting, abdominal distension, diarrhea) causing feeding disorders during enteral nutrition | 396 |
