# Supplementary material for: Risk prediction models for enteral feeding intolerance in critically ill patients: a systematic review
Source: Front Public Health. 2026 Sep 9;14:1900308. doi: 10.3389/fpubh.2026.1900308 (PMC13597850; doi:10.3389/fpubh.2026.1900308)
Supplement: Supplementary file 2 [file Table_2.docx]

**Supplementary Table S2. Model Construction Methods and Predictive Performance**

| First Author | Development Set (n) | Validation Set (n) | Incidence of EFI | Validation Type | Variable Selection | Modeling Method | Validation Method | Predictive Performance | | | |
| --- | --- | --- | --- | --- | --- | --- | --- | --- | --- | --- | --- |
|  |  |  |  |  |  |  |  | Discrimination | AUC | C-index | Calibration |
| Wang et al.[17] | 118 | 118 | 41.5% | a | C, D | F | Q | — | 0.857 (0.779–0.931) | 0.869 | Calibration curve, H–L test |
| Gao[18] | 271 | 271 | 51.7% | a | C, D | F | Q | Sensitivity = 0.814; Specificity = 0.832 | 0.888 (0.850–0.926) | — | Calibration curve, H–L test (P = 0.714) |
| Li et al.[19] | 127 | 127 | 36.2% | a | C, D | F | Q | Sensitivity = 0.891; Specificity = 0.741 | 0.889 (0.821–0.938) | — | Calibration curve |
| Su et al.[20] | 230 | 230 | 35.2% | a | C, D | F | Q | Sensitivity = 0.933; Specificity = 0.743 | 0.879 (0.811–0.947) | — | Calibration curve, H–L test (P = 0.683) |
| Sun et al.[21] | 282 | 282 | 37.9% | a | C, D | F | Q | — | 0.794 (0.741–0.847) | — | Calibration curve, H–L test (P = 0.659) |
| Hu et al.[22] | 124 | 124 / 71 | 44.1% | a, b | C, D | F, J, G, K, L* | T | — | 0.82 (0.74–0.90) | — | Calibration curve |
| Lu et al.[23] | 203 | 203 | 37.9% | a | C, D | F | Q | — | 0.70 (0.63–0.77) | — | Calibration curve |
| Dong et al.[24] | 246 | 246 / 92 | 39.4% | a, b | C, D | F | Q | Sensitivity = 0.818; Specificity = 0.680 | 0.88 (0.811–0.948) | — | H–L test (P = 0.901) |
| Liu et al.[25] | 118 | 118 / 100 | 43.22% | a, b | C, D | F | Q | Sensitivity = 0.925; Specificity = 0.872 | — | 0.879 | Calibration curve |
| Lu et al.[26] | 342 | 342 / 109 | 41.2% | a, b | C, D | F | Q | Sensitivity = 0.881; Specificity = 0.779 | 0.901 (0.869–0.933) | — | Calibration curve, H–L test (P = 0.611) |
| Pan et al.[27] | 114 | 114 / 61 | 47.2% | a, b | E, D | F | R | Sensitivity = 0.803; Specificity = 0.838 | 0.869 (0.810–0.928) | — | Calibration curve, H–L test (P = 0.692) |
| Wang et al.[28] | 628 | 628 / 143 | 49.0% | a, b | C, D | F | Q | — | 0.879 (0.823–0.936) | — | Calibration curve |
| Zang et al.[29] | 246 | 246 / 105 | 41.9% | a, b | C, D | F | R | Sensitivity = 0.818; Specificity = 0.680 | 0.793 (0.735–0.851) | — | Calibration curve, H–L test (P = 0.901) |
| Zhu et al.[30] | 140 | 140 | 49.3% | a | C, D | F | Q | Sensitivity = 0.875; Specificity = 0.833 | 0.906 (0.783–1.000) | — | Calibration curve |
| Raphaeli et al.[31] | 1584 | 1584 | 61.3% | a | C | M, H, G, N, K*, F, P | S | Specificity = 0.77 | 0.710 (0.670–0.740) | — | — |
| Pu et al.[32] | 310 | 360 | 35.0% | a, b | C, D | F | R | Sensitivity = 0.922; Specificity = 0.667 | 0.864 (0.827–0.900) | — | Calibration curve, H–L test (P = 0.098) |
| Chen et al.[33] | 160 | 160 | 26.25% | a | C, D | F | Q | Sensitivity = 0.889; Specificity = 0.766 | 0.904 (0.864–0.944) | — | Calibration curve, H–L test (P = 0.158) |
| Ding et al.[34] | 339 | 146 | 38.4% | a | E, D | F*, H, M, G, I | R | — | 0.88 | — | Calibration curve |
| Liu et al.[35] | 118 | 82 | 41.0% | a | C, D | F | Q | Sensitivity = 0.810; Specificity = 0.820 | 0.852 (0.797–0.881) | — | Calibration curve, H–L test (P = 0.604) |
| Wang et al.[36] | 390 | 97 | 35.9% | a | C, D | F, G*, I | R | — | 0.99 | — | — |
| Wang et al.[37] | 156 | 156 / 102 | 35.9% | a, b | C, D | F | Q | — | 0.966 (0.943–0.989) | 0.993 | Standard curve |
| Wei et al.[38] | 170 | 170 | 30.58% | a | C, D | F | Q | Sensitivity = 0.904; Specificity = 0.670 | 0.866 (0.807–0.925) | — | Calibration curve, H–L test (P = 0.937) |
| Yang et al.[39] | 199 | 199 | 46.7% | a | E, D | F | Q | Sensitivity = 0.849; Specificity = 0.839 | 0.885 (0.833–0.933) | — | Calibration curve |
| Yang et al.[40] | 215 | 92 | 49.2% | a | E, D | F | Q | Sensitivity = 0.792; Specificity = 0.908 | 0.907 (0.867–0.947) | 0.831 | Calibration curve, H–L test (P = 0.889) |
| Yu et al.[41] | 272 | 124 | 35.86% | a | C, D | F*, G, I | R | Sensitivity = 0.997; Specificity = 0.998 | 0.943 | — | — |

a=Internal validation set; b=External validation set; C=Univariate analysis; =Multivariate analysis; E=LASSO regression; F=Logistic regression; G=Random forest; H=Decision tree; I=Support vector machine; J=Naive Bayesian; K=Gradient boosting tree; L=Deep learning; M=K-nearest neighbor; N=XGBoost; P=AdaBoost; Q=Bootstrap validation; R=Random split validation; S=10-fold cross-validation; T=Five-fold cross-validation; *=Optimal model
