# Supplementary material for: Risk prediction models for enteral feeding intolerance in critically ill patients: a systematic review
Source: Front Public Health. 2026 Sep 9;14:1900308. doi: 10.3389/fpubh.2026.1900308 (PMC13597850; doi:10.3389/fpubh.2026.1900308)
Supplement: Supplementary file 3 [file Table_3.docx]

**Supplementary Table S3. Risk of bias and applicability assessment of risk prediction models**

| Study | Bias Assessment | | | | | Applicability Assessment | | | |
| --- | --- | --- | --- | --- | --- | --- | --- | --- | --- |
|  | Study Participants | Predictive Factors | Outcome | Statistical Analysis | Overall Bias Risk | Study Participants | Predictive Factors | Outcome | Overall Applicability Assessment |
| Wang et al.[17] | ① | ② | ① | ① | ① | ④ | ④ | ④ | ④ |
| Gao[18] | ① | ② | ① | ① | ① | ④ | ④ | ④ | ④ |
| Li et al.[19] | ① | ② | ① | ① | ① | ④ | ④ | ④ | ④ |
| Su et al.[20] | ① | ② | ① | ① | ① | ④ | ④ | ④ | ④ |
| Sun et al.[21] | ① | ② | ① | ① | ① | ④ | ④ | ④ | ④ |
| Hu et al.[22] | ① | ② | ① | ① | ① | ④ | ④ | ④ | ④ |
| Lu et al.[23] | ① | ② | ① | ① | ① | ④ | ④ | ④ | ④ |
| Dong et al.[24] | ① | ② | ① | ① | ① | ④ | ④ | ④ | ④ |
| Liu et al.[25] | ② | ② | ① | ① | ① | ④ | ④ | ④ | ④ |
| Lu et al.[26] | ① | ② | ① | ① | ① | ④ | ④ | ④ | ④ |
| Pan et al.[27] | ① | ② | ① | ① | ① | ④ | ④ | ④ | ④ |
| Wang et al.[28] | ① | ② | ① | ① | ① | ④ | ④ | ④ | ④ |
| Zang et al.[29] | ② | ② | ① | ① | ① | ④ | ④ | ④ | ④ |
| Zhu et al.[30] | ① | ② | ① | ① | ① | ④ | ④ | ④ | ④ |
| Raphaeli et al.[31] | ① | ② | ① | ① | ① | ④ | ④ | ④ | ④ |
| Pu et al.[32] | ① | ② | ① | ① | ① | ④ | ④ | ④ | ④ |
| Chen et al.[33] | ① | ② | ① | ① | ① | ④ | ④ | ④ | ④ |
| Ding et al.[34] | ① | ② | ① | ① | ① | ④ | ④ | ④ | ④ |
| Liu et al.[35] | ① | ② | ① | ① | ① | ④ | ④ | ④ | ④ |
| Wang et al.[36] | ① | ② | ① | ① | ① | ④ | ④ | ④ | ④ |
| Wang et al.[37] | ① | ② | ① | ① | ① | ④ | ④ | ④ | ④ |
| Wei et al.[38] | ① | ② | ① | ① | ① | ④ | ④ | ④ | ④ |
| Yang et al.[39] | ① | ② | ① | ① | ① | ④ | ④ | ④ | ④ |
| Yang et al.[40] | ① | ② | ① | ① | ① | ④ | ④ | ④ | ④ |
| Yu et al.[41] | ① | ② | ① | ① | ① | ④ | ④ | ④ | ④ |

① = high risk of bias; ② = low risk of bias; ③ = high concern regarding applicability; ④ = low concern regarding applicability
